# Frailty and risk of adverse outcomes among community-dwelling older adults in China: a comparison of four different frailty scales

**DOI:** 10.3389/fpubh.2023.1154809

**Published:** 2023-05-10

**Authors:** Fei Qin, Yanfei Guo, Ye Ruan, Zhezhou Huang, Shuangyuan Sun, Shuna Gao, Jinghong Ye, Fan Wu

**Affiliations:** ^1^School of Public Health, Fudan University, Shanghai, China; ^2^Division of Chronic Non-communicable Disease and Injury, Shanghai Municipal Center for Disease Control and Prevention, Shanghai, China; ^3^School of Public Health and Community Medicine, Institution of Medicine, University of Gothenburg, Sahlgrenska Academy, Gothenburg, Sweden; ^4^Department of Chronic Non-communicable Disease, Shanghai Huangpu Center for Disease Control and Prevention, Shanghai, China; ^5^Department of Chronic Non-communicable Disease, Shanghai Hongkou Center for Disease Control and Prevention, Shanghai, China

**Keywords:** frailty, predictive accuracy, older Chinese, population-based, longitudinal study, adverse outcomes

## Abstract

**Background:**

Data on which frailty scales are most suitable for estimating risk in Chinese community populations remain limited. Herein we examined and compared four commonly used frailty scales in predicting adverse outcomes in a large population-based cohort of Chinese older adults.

**Methods:**

A total of 5402 subjects (mean age 66.3 ± 9.6 years, 46.6% male) from the WHO Study on global AGEing and adult health (SAGE) in Shanghai were studied. Frailty was measured using a 35-item frailty index (FI), the frailty phenotype (FP), FRAIL, and Tilburg Frailty Indicator (TFI). Multivariate logistic regression models were performed to evaluate the independent association between frailty and outcomes including 4-year disability, hospitalization, and 4- and 7-year all-cause mortality. The accuracy for predicting these outcomes was determined by evaluating the area under the curve (AUC). The prevalence of frailty, sensitivity, and specificity were calculated using our proposed cut-off points and other different values.

**Results:**

Prevalence of frailty ranged from 4.2% (FRAIL) to 16.9% (FI). FI, FRAIL and TFI were comparably associated with 4-year hospitalization, and 4- and 7-year mortality (adjusted odds ratios [aORs] 1.44–1.69, 1.91–2.22 and 1.85–2.88, respectively). FRAIL conferred the greatest risk of 4-year disability, followed by FI and TFI (aOR 5.55, 3.50, and 1.91, respectively). FP only independently predicted 4- and 7-year mortality (aOR 1.57 and 2.21, respectively). AUC comparisons showed that FI, followed by TFI and FRAIL, exhibited acceptable predictive accuracy for 4-year disability, 4- and 7-year mortality (AUCs 0.76–0.78, 0.71–0.71, 0.65–0.72, respectively), whereas all scales poorly predicted 4-year hospitalization (AUCs 0.53–0.57). For each scale, while specificity estimates (85.3–97.3%) were high and similar across all outcomes, their sensitivity estimates (6.3–56.8%) were not sufficient yet. Prevalence of frailty, sensitivity, and specificity varied considerably when different cut-off points were used.

**Conclusion:**

Frailty defined using any of the four scales was associated with an increased risk of adverse outcomes. Although FI, FRAIL and TFI exhibited fair-to-moderate predictive accuracy and high specificity estimates, their sensitivity estimates were not sufficient yet. Overall, FI performed best in estimating risk, while TFI and FRAIL were additionally useful, the latter perhaps being more applicable to Chinese community-dwelling older adults.

## 1. Introduction

Frailty describes a non-specific state reflecting cumulative declines in multiple physiological systems with aging, leading to decreased resilience to stressors (1). Routine screening for frailty among older adults has been called for (2); however, no uniformly accepted operational definition for frailty is currently available (1, 3). Most commonly, frailty has been operationalized as the frailty phenotype (FP) based on the biologic syndrome model proposed by Fried and colleagues (4). In comparison, the frailty index (FI) was developed as a scale of deficit accumulation model to measure the cumulative burden of, for example, diseases, symptoms, and conditions (5). Furthermore, the FRAIL, proposed by the International Academy of Nutrition Health and Aging (IANA) and developed as a simple measure that combines elements from both the FI and FP models, as well as the Tilburg Frailty Indicator (TFI), described in line with an integral conceptual model of frailty by a group of Canadian researchers based on interview-based questions, are also frequently used (6).

A substantial number of frailty scales including the four above, irrespective of the frailty definition used, have been shown to predict a variety of adverse outcomes (7), while in practice choosing a scale is sometimes arbitrary, e.g., based solely on available data, yet how frailty is conceptualized affects aging research. For example, given that multiple frailty-related health outcomes, such as disability, hospitalization and all-cause death, can affect lots of people, it is crucial to determine whether one frailty scale has advantages over others in identifying and predicting high-risk groups. As a result, comparisons between frailty scales in estimating risk have been performed but the results are still controversial (8–11), partially attributed to differences in study populations, settings, outcomes, follow-up periods, and even the criteria selected to operationalize frailty. This highlights the need for careful examination of the predictive properties of frailty scales for health outcomes in different populations and settings for subsequent research.

In China, the largest developing country with a rapidly aging population, several longitudinal cohorts have explored frailty and risk of adverse outcomes, including the Chinese Longitudinal Health and Longevity Study (CLHLS) (12, 13), the Beijing Longitudinal Study of Aging (BLSA) (14, 15) and the Rugao Longevity and Aging Study (RuLAS) (16, 17), the majority of which focus on the relationship between FI and/or FP and mortality. In an earlier longitudinal study of 4,000 Hong Kong Chinese aged 65 and older (8), the FRAIL scale was found comparable with FI and FP in the prediction of mortality and physical limitations over 4 years of follow-up. Recently, another longitudinal study of 302 Chinese hospitalized older patients (median age 86 years) found that four different frailty scales showed similar performance in predicting 1-year in-hospital mortality (18). However, data on the relationship between multiple frailty scales and adverse outcomes are still limited, and even to date, no longitudinal studies have compared multiple frailty scales in predicting long-term health outcomes within the same timeframe in the same mainland Chinese community-dwelling population, making it difficult to determine which frailty scale should be used as an outcome measure.

To fill the above gap, we analyzed the results of a population-based cohort study involving 5,402 Chinese community-dwelling adults aged 50 and older, in which four frailty scales were explored and compared. Some related frailty scales were not included because they are more focused on relatively small scopes (e.g., timed-up-and-go test, scarcopenia) or are less directly applicable to population-based settings (e.g., laboratory-based biomarkers), or cannot be constructed using the present data (e.g., Clinical Frailty Scale). In this longitudinal study, we sought to examine and compare the utility of four commonly used frailty scales adapted from existing frailty approaches in identifying frailty, together with their ability to predict several adverse outcomes (4-year disability, hospitalization, and 4- and 7-year all-cause mortality), for the sake of identifying at-risk groups and potentially reversing established frailty status.

## 2. Materials and methods

### 2.1. Study sample

**Figure 1:**
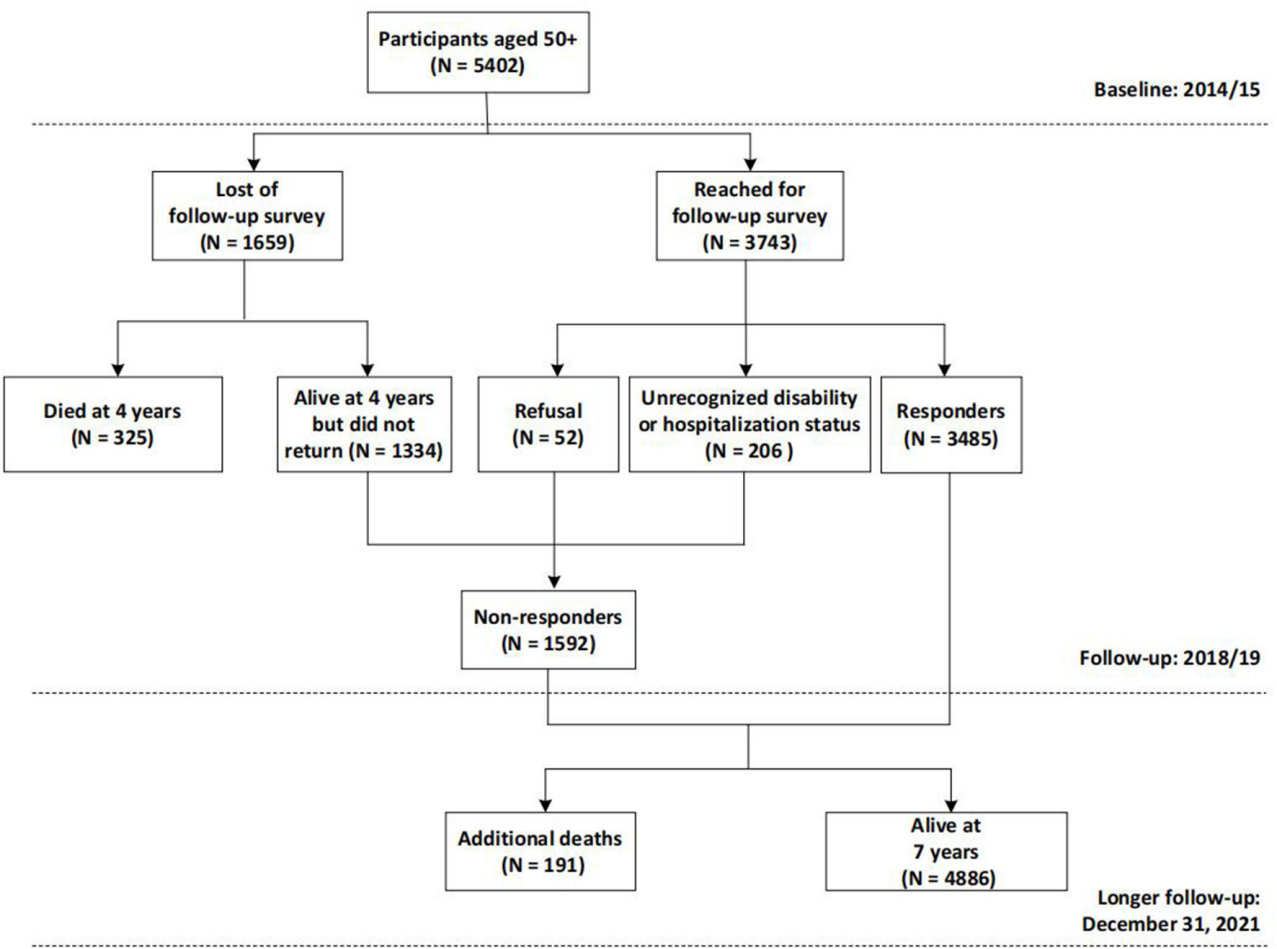
Flow chart of the selection of subjects for the cohort study.

Participants were drawn from a large ongoing population-based cohort study, the WHO Study on global AGEing and adult health (SAGE) in Shanghai. Details concerning the SAGE have been previously described (19). Briefly, SAGE is a longitudinal study on the health and wellbeing of adults aged 50 and older in six low- and middle-income countries (LMICs): China, Ghana, India, Mexico, Russian and South Africa. In China, the study was constructed including wave 1, implemented in 2009/10, wave 2 in 2014/15 and wave 3 in 2018/19. We enlarged the sample size of SAGE in Shanghai, China to obtain a sub-state representative sample using the same multistage clustered sampling method and survey assessment. In particular, wave 2 served as the baseline and wave 3 as the follow-up of the current study, as they contained a more comprehensive set of assessments. A longer follow-up through December 31, 2021 was additionally conducted to ascertain the participants' survival status. At baseline (2014/15), 5,402 community dwellers aged 50 and older were recruited from five districts of Shanghai, China and included in the analysis for mortality. After 4 years, 5,077 subjects (325 had died) were invited to undergo the follow-up assessment, while 1,592 were excluded (1334 did not return, 52 declined, and 206 had unrecognized disability or hospitalization); leaving 3,485 participants eligible for the analysis for disability and hospitalization (Figure 1). Comparisons of the non-responders with respondents in terms of baseline age, sex, and frailty status were conducted (see Supplementary material S1) and results suggested that the issue of the representativeness should not represent a potential bias, despite a response rate of 68.6%.

### 2.2. Frailty scales

The four frailty scales are briefly described below. An overview of all items constructed in each scale can be found in Supplementary material S2. In particular, to maximize the use of available data, a scale was included in subsequent analyses if no more than 20% of all items were missing (11); meanwhile, missing items for FP, FRAIL, and TFI were imputed with 0 (having no this deficit), whereas no substitution procedure was required for FI because of its distinctive derivation method used in this study.

Frailty index (FI). The FI is based on the cumulative deficit model that identifies frailty based on a range of variables across multiple domains, such as diseases, symptoms, and conditions, collectively referred to as deficits. It has been suggested that an index with 30–40 variables is sufficiently accurate for predicting adverse outcomes. Following a standard procedure (20), we created a 35-item FI comprising 7 components: self-rated health, medically diagnosed conditions (9 items), medical symptoms (6 items), functional activities assessments (11 items), cognitive function assessments (5 items), body mass index (BMI), and physical performance tests (2 items). The included variables were dichotomous, ordinal or continuous. Dichotomous variables were coded as 0 as non-deficit and 1 being a deficit; ordinal and continuous variables were converted as a certain proportion of the deficit. For each participant, these deficits were summed up and then divided by the total possible deficit to derive the FI. In line with previous SAGE studies in Chinese populations (21, 22), individuals with an index of 0.20 or greater were considered to be frail.

Frailty phenotype (FP). The FP was constructed using an adapted phenotypic definition based on the criteria of five components proposed by Fried et al. (4): slowness, weight loss, low grip strength, exhaustion, and low physical activity. It has been previously operationalized in SAGE (23–25), and the same criteria were applied in this study. In short, exhaustion and physical activity are self-report questions, while slowness, weight loss and low grip strength are performance-based measures. Likewise, participants were classified as frail if 3 or more criteria were present.

FRAIL scale. We used an adaption of the IANA FRAIL scale (26), which considers deficits accumulated in five domains: fatigue, resistance, ambulation, illness, and loss of weight. FRAIL has not been explored in SAGE before. Fatigue was measured on a 5-point Likert scale by asking respondents whether they had enough energy for daily activities. This criterion was considered present if participants answered “Not at all” or “A little”. Resistance and ambulation were obtained by asking “Do you have any difficulty standing for long periods” and “Do you have any difficulty walking 1 kilometer”, respectively. Resistance or ambulation was considered present if subjects answered “Severe” or “Extreme/Cannot do”. Participants were classified as ill if they had 5 or more out of 9 self-reported chronic diseases including diabetes mellitus, stroke, cataracts, angina pectoris, arthritis, asthma, chronic lung disease, depression and hypertension. The weight loss criterion was ascertained based on the lowest quintile of BMI. Individuals with 3 or more criteria were recognized as frail.

Tilburg Frailty Indicator (TFI). The TFI, developed as an integral conceptual model of frailty, comprises two subscales (27). One subscale addresses the determinants of frailty such as socio-demographics, the latter addresses the level of frailty across physical (8 items), psychological (4 items) and social domains (3 items), and is used in this study, yet it has not previously been explored in SAGE. Memory problems were measured using a delayed recall memory test and the cut-off point was the worst-performing 10th centile. Anxiety was assessed using a question about irritability, and coping was obtained by asking the individual “How often have you found that you could not cope with all the things that you had to do?”, and was considered present if people answered, “Fairly often” or “Very often”. Social deficits were assessed by asking the individual “What is the total number of people who live in your household?”, “How satisfied are you with your personal relationships” and “Were you supported for the last time when you needed it?”. Theoretical scores of the TFI range from 0 to 15, with a score of 5 or greater defining frailty.

### 2.3. Outcome measures

Outcome measures were new development of disability, hospitalization at 4 years, and 4- and 7-year all-cause mortality.

Disability was assessed both during 2014/15 and 2018/19 using eight activities of daily living (ADL) tasks (moving around, bathing, dressing, maintaining appearance, getting up from lying down, eating, toileting, and controlling urine) (28). For each ADL task, participants were asked, “Do you have difficulty in” performing the task in the preceding 30 days? The response was in a Likert scale format ranging from “None” to “Extreme/Cannot”. Respondents were considered to have ADL disability if they reported severe or extreme difficulties in performing at least one of the eight tasks listed above; then, the onset of a new disability was defined as a newly identified disability during 2018/19. For hospitalization, participants were asked “whether you had stayed at least overnight in a hospital since the last interview, i.e., in the prior 4 years?” during 2018/2019. Finally, 4- and 7-year all-cause mortality was determined by linking data to the Shanghai Death Registry during 2018/2019 and on December 31, 2021, respectively.

### 2.4. Covariates

Using the literature on disability, hospitalization, and mortality in older adults as a guide (9, 16, 17), commonly cited risk factors were selected as potential covariates and then identified in the dataset. Hence, covariates included age, sex (male or female), marital status (partnered [married/cohabiting], not partnered [separated/divorced/widowed or never married]), educational level achieved (no education, less than primary, primary, secondary or higher), smoking status (never smoked, current smoker or former smoker) and body mass index (BMI). For smoking status, respondents were first asked “Have you ever smoked tobacco or used smokeless tobacco?” Those who answered “No” were classified as never smoked, while those who answered “Yes” were then asked “Do you currently use (smoke, sniff or chew) any tobacco products such as cigarettes, cigars, pipes, chewing tobacco or snuff?” If the respondents answered “Yes” again, they were classified as current smokers, otherwise they were classified as former smokers. Measured height and weight were used to calculate a standard BMI (calculated as weight in kilograms divided by height in meters squared).

### 2.5. Statistical analysis

Descriptive statistics were presented as either means (standard deviations) or frequencies (percentages), with comparisons between four different outcome groups using *t*-tests/Wilcoxon rank-sum tests or chi-square tests, as appropriate. Logistic regression models were measured to investigate the association of dichotomized frailty status [frail, non-frail (reference)] identified by each scale with adverse outcomes, with results reported as odds ratios (ORs) and 95% confidence intervals (CIs). All regression models were performed and adjusted for the same multiple covariates above (fixed model). For each outcome, a receiver operator characteristic (ROC) curve based on the continuous scores of each scale was created and the area under the curve (AUC) was calculated with their corresponding 95% CIs to assess the unadjusted ability of each scale to differentiate between the frail and non-frail participants; AUCs between frailty scales were then compared using Wilcoxon tests to ascertain if there is a statistical difference. The prevalence of frailty, sensitivity and specificity for each scale and for each outcome were also calculated using our proposed cut-off points as well as those points one above and one below our proposed values (0.05 for the FI). We used the following acceptable minimum thresholds: ≥0.60 for AUC (29), ≥0.8 for sensitivity (30), and ≥0.6 for specificity (30). Statistical analyses were performed using the SAS software (version 9.4, SAS Institute, Inc., Cary, NC), and a 2-sided *p* < 0.05 was considered statistically significant.

## 3. Results

The baseline characteristics of the cohort are described in Table 1. Of 5,402 participants, 2,515 (46.6%) were men and 2,887 (53.4%) were women. The participants ranged in age from 50 to 97 years, with a mean age of 66.3 (SD: 9.6) years. Most (85.8%) of the participants were currently partnered, while few (19.4%) were illiterate. Approximately one-quarter (24.9%) of the participants were current smokers. The prevalence of frailty varied between scales: FRAIL, 4.2%; TFI, 7.3%; FP, 12.6%; FI, 16.9%, although between 103 (1.9%) and 244 (4.5%) participants were unable to be assessed by the four scales due to missing data (>20% items) (see Supplementary material S3).

**Table 1:** Baseline characteristics and the difference between participants with and without adverse outcomes.

**Variable**	**All (*n* = 5402)**	**4-year disability (*****n =*** **125)**		**4-year hospitalization (*****n =*** **720)**		**4-year mortality (*****n =*** **325)**		**7-year mortality (*****n =*** **516)**	
		**Value**	*p* ^†^	**Value**	*p* ^†^	**Value**	*p* ^†^	**Value**	*p* ^†^
**Mean (SD)**									
Age (years)	66.3 (9.6)	72.6 (9.1)	<0.001	67.1 (8.4)	<0.001	78.3 (9.7)	<0.001	78.5 (9.8)	<0.001
BMI (kg/m^2^)	19.5 (3.0)	19.0 (2.9)	0.026	19.6 (3.1)	0.730	18.8 (3.4)	<0.001	18.8 (3.4)	<0.001
**Number (%)**									
Sex (male)	2515 (46.6)	56 (44.8)	0.856	357 (49.6)	0.016	170 (52.3)	0.032	265 (51.4)	0.022
Marital status			<0.001		0.507		<0.001		<0.001
Not partnered	767 (14.2)	28 (22.4)		93 (12.9)		116 (35.7)		183 (35.5)	
Partnered	4635 (85.8)	97 (77.6)		627 (87.1)		209 (64.3)		333 (64.5)	
**Educational level**			<0.001		<0.001		<0.001		<0.001
No education	1049 (19.4)	48 (38.4)		166 (23.1)		129 (39.7)		198 (38.4)	
Less than primary	718 (13.3)	20 (16.0)		128 (17.8)		49 (15.1)		68 (13.2)	
Primary	1026 (19.0)	24 (19.2)		157 (21.8)		49 (15.1)		84 (16.3)	
Secondary	1534 (28.4)	24 (19.2)		168 (23.3)		49 (15.1)		90 (17.4)	
Higher	1075 (19.9)	9 (7.2)		101 (14.0)		49 (15.1)		76 (14.7)	
**Smoking status**			0.475		0.904		0.440		<0.001
Never smoked	3825 (70.8)	92 (73.6)		518 (72.0)		235 (61.7)		334 (64.7)	
Former smoker	232 (4.3)	7 (5.6)		29 (4.0)		17 (7.0)		35 (6.8)	
Current smoker	1345 (24.9)	26 (20.8)		173 (24.0)		73 (31.3)		147 (28.5)	
**Frailty status (frail)** ^*^									
FI	888 (16.9)	64 (51.2)	<0.001	150 (21.0)	<0.001	154 (55.0)	<0.001	258 (56.8)	<0.001
FP	648 (12.6)	26 (20.8)	0.002	109 (15.2)	0.002	63 (30.6)	<0.001	117 (34.1)	<0.001
FRAIL	224 (4.2)	32 (25.6)	<0.001	45 (6.3)	<0.001	56 (19.5)	<0.001	91 (19.6)	<0.001
TFI	385 (7.3)	28 (22.4)	<0.001	72 (10.0)	<0.001	75 (27.3)	<0.001	117 (26.2)	<0.001

SD, Standard Deviation; BMI = Body Mass Index; FI, Frailty Index; FP, Frailty Phenotype; TFI, Tilburg Frailty Indicator.

† *p* value for comparison of difference between adverse outcome groups: t-test or Wilcoxon rank-sum test (depending on distribution) for continuous variables, Chi-square test for categorical variables.

* Due to missing data, small differences between n and numbers of participants reported for each scale can occur.

After 4 years of follow-up, 325 (6.0%) of 5,402 participants had died; of 3,485 responders, 125 (3.6%) developed a new disability and 720 (20.7%) reported one or more new hospitalizations, respectively. Additionally, after a longer 7-year follow-up, a total of 516 participants died, resulting in a greater mortality rate of 9.6%. Compared with their counterparts, those with adverse outcomes generally were older, less educated, and frailer using any scale at baseline (all *p* < 0.01) (Table 1).

For each scale of interest, Table 2 details the risk of selected adverse outcomes in frail compared to non-frail participants. Multivariate logistic regression found frailty by any of the FI, FP, FRAIL, and TFI scales to be a strong predictor of all-cause mortality (all *p* < 0.05), with adjusted ORs of 2.22, 1.57, 1.91, and 1.94 for 4-year mortality, 2.88, 2.21, 2.29, and 1.85 for 7-year mortality, respectively. The risk of 4-year disability was also associated with frailty by any scale (except FP), but the association was stronger for FRAIL (adjusted OR 5.55) than for either FI (adjusted OR 3.50) or TFI (adjusted OR 1.91) (all *p* < 0.05). Frailty by these three scales was additionally comparably associated with 4-year hospitalization (adjusted ORs 1.44–1.69, all *p* < 0.05). Of note, the independent association with risk of 4-year either disability or hospitalization did not reach statistical significance for FP (both *p* >0.05).

**Table 2:** Comparison of adverse outcomes between baseline frail and non-frail participants during follow-up.

	**4-year disability**		**4-year hospitalization**		**4-year mortality**		**7-year mortality**	
	**Adjusted**^a^ **OR (95% CI)**	***p***	**Adjusted**^a^ **OR (95% CI)**	***p***	**Adjusted**^a^ **OR (95% CI)**	***p***	**Adjusted**^a^ **OR (95% CI)**	***p***
FI	3.50 (2.19, 5.61)	<0.001	1.44 (1.11, 1.88)	0.006	2.22 (1.42, 3.48)	<0.001	2.88 (2.03, 4.08)	<0.001
FP	1.06 (0.64, 1.76)	0.816	1.15 (0.89, 1.50)	0.278	1.57 (1.12, 2.20)	0.008	2.21 (1.53, 3.20)	<0.001
FRAIL	5.55 (3.20, 9.62)	<0.001	1.64 (1.07, 2.52)	0.024	1.91 (1.05, 3.46)	0.035	2.29 (1.43, 3.66)	<0.001
TFI	1.91 (1.12, 3.27)	0.018	1.69 (1.21, 2.37)	0.002	1.94 (1.18, 3.20)	0.010	1.85 (1.24, 2.76)	0.003

FI, Frailty Index; FP, Frailty Phenotype; TFI, Tilburg Frailty Indicator; OR, Odds Ratio; CI, Confidence Interval.

a Logistic regression models adjusted for baseline age, sex, educational level, marital status, BMI, and smoking status.

**Figure 2:**
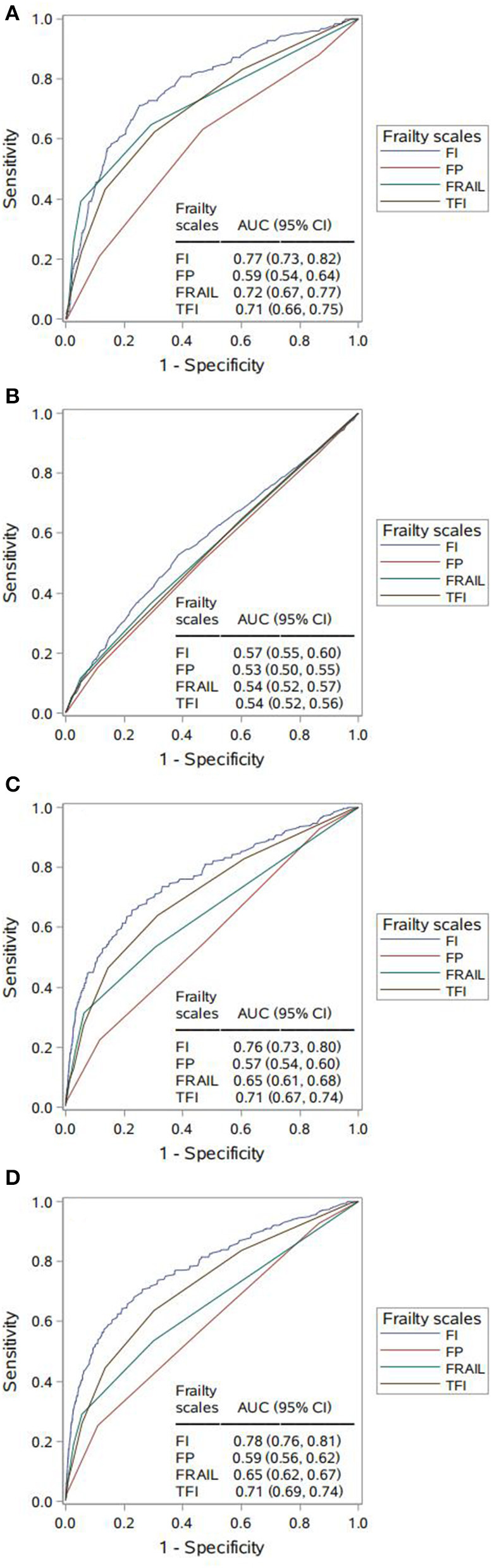
Comparing area under the curve (AUC) for the four frailty scales per adverse outcome. **(A)** 4-year disability. AUC contrasts: Frailty Index (FI) vs. Frailty Phenotype (FP), *p* < 0.001; FI vs. FRAIL, *p* = 0.018; FI vs. Tilburg Frailty Indicator (TFI), *p* = 0.002; FP vs. FRAIL, *p* < 0.001; FP vs. TFI, *p* < 0.001; FRAIL vs. TFI, *p* = 0.549. **(B)** 4-year hospitalization. AUC contrasts: FI vs. FP, *p* < 0.001; FI vs. FRAIL, *p* = 0.014; FI vs. TFI, *p* = 0.001; FP vs. FRAIL, *p* = 0.135; FP vs. TFI, *p* = 0.256; FRAIL vs. TFI, *p* = 0.722. **(C)** 4-year mortality. AUC contrasts: FI vs. FP, *p* < 0.001; FI vs. FRAIL, *p* < 0.001; FI vs. TFI, *p* < 0.001; FP vs. FRAIL, *p* < 0.001; FP vs. TFI, *p* < 0.001; FRAIL vs. TFI, *p* < 0.001; **(D)** 7-year mortality. AUC contrasts: FI vs. FP, *p* < 0.001; FI vs. FRAIL, *p* < 0.001; FI vs. TFI, *p* < 0.001; FP vs. FRAIL, *p* < 0.001; FP vs. TFI, *p* < 0.001; FRAIL vs. TFI, *p* < 0.001.

We further estimated and compared the predictive accuracy of each scale for each adverse outcome in Figure 2. Per adverse outcome, AUC comparisons showed that the four scales had distinctive predictive accuracy; regarding 4-year disability, 4- and 7-year mortality, FI was more predictive than the other scales (AUC 0.76–0.78), followed by TFI (AUC 0.71) and FRAIL (AUC 0.65–0.72), which performed better than FP (AUC 0.57–0.59) (Figures 2A, C, D). By contrast, all scales had poor accuracy in predicting new hospitalizations at 4-year follow-up, with FI (AUC 0.57) being a better predictor than FRAIL, which was equivalent to both FP and TFI (AUC 0.53–0.54) (Figure 2B).

We also determined the prevalence of frailty as well as the associated sensitivity and specificity based on different cut-off points, as described in Table 3. With our proposed cut-offs in the current study, the prevalence of frailty in this population varied. Regarding the associated diagnostic values, each scale showed high and similar levels of specificity for all outcomes (FI: 85.3–87.7%, FP: 88.2–89.0%, TFI: 93.8–95.0%, FRAIL: 96.7–97.3%). In contrast, sensitivity estimates varied widely within lower ranges: for 4-year disability, 4- and 7-year mortality, each scale showed similar levels of sensitivities, while FI had higher estimates (51.2%, 55.0% and 56.8%, respectively) compared to the other three scales, the latter showed similar sensitivity estimates at lower levels (range 20.8–34.1% for FP, 19.5–25.6% for FRAIL, and 22.4–27.3% for TFI, respectively); for 4-year hospitalization, lowest sensitivity estimates were found across all scales (FI 21.0%, FP 15.2%, FRAIL 6.3%, TFI 10.0%). Furthermore, the sensitivity and specificity for each scale were found to vary considerably when higher or lower cut-off points were applied. For all frailty scales, with increasing levels of frailty, the specificity fell and the sensitivity increased, and yet no scale had both acceptable sensitivity and specificity.

**Table 3:** Prevalence of frailty, sensitivity, and specificity for different cutoffs of each scale for each outcome.

**Frailty scale**	**Cutoff**	Frail [n (%)]^*^	**4-year disability**		**4-year hospitalization**		**4-year mortality**		**7-year mortality**	
			**Sens (%)**	**Spec (%)**	**Sens (%)**	**Spec (%)**	**Sens (%)**	**Spec (%)**	**Sens (%)**	**Spec (%)**
FI	≥ 0.15	1704 (32.4)	72.8	71.7	39.6	72.6	70.0	69.8	71.2	71.3
	**≥0.20**	**888 (16.9)**	**51.2**	**87.3**	**21.0**	**87.7**	**55.0**	**85.3**	**56.8**	**86.9**
	≥ 0.25	560 (10.6)	33.6	92.8	13.0	93.1	45.0	91.3	44.9	92.6
FP	≥ 2	2482 (48.1)	63.2	53.0	51.1	53.3	69.4	52.8	71.7	53.6
	**≥3**	**648 (12.6)**	**20.8**	**88.5**	**15.2**	**89.0**	**30.6**	**88.2**	**34.1**	**89.0**
	≥ 4	26 (0.5)	0	99.6	0.4	99.6	2.9	99.6	3.2	99.7
FRAIL	≥ 2	406 (7.7)	39.2	94.8	11.7	95.0	31.7	93.7	29.5	94.4
	**≥3**	**224 (4.2)**	**25.6**	**97.3**	**6.3**	**97.1**	**19.5**	**96.7**	**19.6**	**97.3**
	≥ 4	78 (1.5)	4.8	99.0	2.1	99.1	7.7	98.9	7.5	99.1
TFI	≥ 4	847 (16.1)	43.2	86.5	19.3	86.7	45.8	85.6	44.4	86.6
	**≥5**	**385 (7.3)**	**22.4**	**94.6**	**10.0**	**95.0**	**27.3**	**93.8**	**26.2**	**94.5**
	≥ 6	171 (3.2)	11.2	97.6	5.2	97.9	12.7	97.3	12.8	97.6

FI, Frailty Index; FP, Frailty Phenotype; TFI, Tilburg Frailty Indicator; Sens, Sensitivity; Spec, Specificity.

The proposed cutoff values used in this study are highlighted in bold.

* Due to missing data, small differences between n and numbers of participants reported for each scale can occur.

## 4. Discussion

To date, this large-scale prospective cohort study has been the first attempt to simultaneously identify and compare the four validated frailty scales for their utility in identifying frailty, together with their ability to predict adverse outcomes in mainland Chinese community dwellers. In this study, we found a low prevalence of frailty as assessed by the FI, FP, FRAIL, and TFI among Chinese community-dwelling older adults. With four frailty scales, frailty was associated with multiple adverse outcomes, including 4-year disability (except FP), hospitalization (except FP), and 4- and 7-year all-cause mortality. However, the four frailty scales showed mixed predictive accuracy as well as associated sensitivity and specificity for the outcomes of interest, indicating that different frailty scales may point to various risks of further adverse outcomes.

In this large representative sample of Chinese community dwellers, we found 4.2% up to 16.9% of Chinese adults aged 50 years or older were frail between the scales. The low frailty prevalence estimates in our cohort are consistent with previous studies (14, 21), although widely varying frailty prevalence estimates have also been observed among community dwellers in LIMICs (31) due to differences in population and the myriad of frailty scales used. By using the most commonly used scales, FI and FP, more than 10% of our cohort fulfilled the criteria for frailty, whereby only 7.3% or 4.2% would have been frail by TFI or FRAIL, respectively. In a European study (32), albeit among hospitalized patients, a higher proportion of the cohort was considered frail on FI and TFI compared with the FRAIL scale. We found that, unlike the multidimensional FI and TFI, FRAIL did not capture psychological and social components, which may have contributed to its lower detection rates of frailty. In addition, compared with FRAIL and TFI, FP was largely guided by physical performance, including walking speed and grip strength, yielding a higher prevalence estimate of frailty.

Previous studies have simultaneously validated the studied scales longitudinally in different European populations. However, these results may not be generalizable because the exposure pattern and disease spectrum of Europeans are quite different from those of the Chinese, especially for older adults. FI, FRAIL, and TFI demonstrated independent predictive validity against all outcomes of interest in this study, suggesting that they could identify high-risk Chinese older adults, as measured by 4-year disability, hospitalization, and 4- and 7-year all-cause mortality. The results are consistent with those of other studies (10, 11, 18), although most of them focus on mortality. Furthermore, while these three scales were comparably associated with 4-year hospitalization and 4- and 7-year mortality, their strengths of association with 4-year disability were different; FRAIL conferred the greatest risk, followed by FI and TFI (adjusted OR 5.55, 3.50 and 1.91, respectively, all *p* < 0.05). Notably, there was no evidence of an independent association between FP and 4-year disability or hospitalization in the multivariate analysis, which contrasted with previously published data (33, 34). This discrepancy may be attributable to the partially modified components as well as different covariates adjustments used in our study. Nevertheless, we found that FP was independently associated with mortality, even allowing for different follow-up periods.

Notably, differences were also evident between our unadjusted logistic models where the predictive accuracy (estimated using AUC) of FI was significantly higher than that of either FRAIL or TFI, all of which offered an advantage over FP. This is perhaps unsurprising, as multidimensional geriatric measures may provide better identification of frailty-related outcomes than a unidimensional index exclusively focused on muscular fitness. Moreover, regarding 4-year disability and 4-and 7-year mortality, AUCs for FI, FRAIL and TFI were acceptable and slightly higher than those of other population-based studies (16, 33, 35); all studied scales, however, were least able to discriminate 4-year hospitalization, which was consistent with these studies. For example, FI, FP, and FRAIL were investigated in the African American Health (AAH) cohort (10), and the findings showed AUCs of 0.69, 0.66 and 0.68 respectively, for 9-year disabilities, 0.64, 0.57 and 0.53 for 9-year mortality. A retrospective study in the Australian Longitudinal Study of Aging (ALSA) (33) revealed that both FI and FP had a low ability to discriminate hospitalization (AUC <0.6). In short, we demonstrated that in this Chinese older population, FI, FRAIL, and TFI are useful predictors for predicting 4-year disability and 4- and 7-year all-cause mortality, whereas none of the four scales should be used as the sole tool for screening for risk of hospitalization.

Another interesting finding in our study was that although the AUC for adverse outcomes was different between the scales, similar performances were found in their diagnostic values of sensitivity and specificity. The high and similar specificity estimates indicated that all frailty scales can be comparably useful in identifying non-frail participants in those without adverse outcomes. Correspondingly, sensitivity estimates for different frailty scales varied within low ranges; regarding 4-year disability and 4- and 7-year mortality, the FI showed similar levels of sensitivity (range 51.2–56.8%), and the other three scales showed similar sensitivity at poor levels (range 20.8–34.1% for FP, 19.5–25.6% for FRAIL, and 22.4–27.3% for TFI, respectively), and lowest sensitivities were found for each scale in the prediction of 4-year hospitalization (range 6.3-21.0%). These findings were consistent with those of a previous study conducted in an older Australian population (33), and the low sensitivity suggested a lack of identification of frail participants at risk for adverse outcomes with our proposed cut-off points. Recently, a similar population-based study (9) of Dutch community-dwelling older people examined the predictive accuracy of FI, FP, and TFI for adverse outcomes including death, hospitalization, and ADL dependency, with a 2-year follow-up. It reported comparable specificity values for FP 79.6–86.2%; however, the sensitivity values were slightly better: 24.7–44.5%. For TFI, the Dutch study reported higher sensitivity values of 70.5–80.6% than those of 10.0–27.3% in the present study, while its specificity values were lower (36.5–45.7%). In addition, compared to the FI used in the Dutch study with a cut-off value of 0.25, the FI with a lower cut-off value of 0.2 used in our Chinese population was found to have higher sensitivity (except for hospitalization) and specificity. A possible reason for this disparity is that our study used the cut-off points proposed by the original authors for FP, FRAIL, and TFI, however, these cutoffs may not be sensitive enough to detect small changes in frailty status when applied to the Chinese population, especially given that we observed higher sensitive values when lower cut-offs were applied (Table 3). Previous studies (36, 37) on the validation of frailty scales also suggested that for the TFI and FRAIL scales used in Chinese community-dwelling older adults, the optimal cut-off points for frailty were 4 and 2, respectively, which were slighter lower than the original values. Additionally, we speculate that different components of the scales and definitions of the outcomes may also have contributed to this disparity.

In general, good frailty scales should have high predictive ability and sensitivity, the latter of which will be the most relevant criterion, as higher sensitivity means a lower risk of withholding additional investigation and, if available, possible treatments from people who might need it. In the current study, while the scales (except FP) showed distinctively acceptable predictive ability, none of them had both acceptable sensitivity and specificity, nor when the cutoffs were increased or decreased. Therefore, we recommend that choosing a scale will greatly depend on the purpose and setting for frailty assessment. From this perspective, FI, TFI, and FRAIL are useful predictors and frailty screening tools for the development of disability and death in intervention programs such as being inclusion criteria for clinical trials, in which higher specificity is preferred over sensitivity, as it is preferable to correctly identify frail individuals, although some frail individuals will be missed. When screening for geriatric conditions in primary care, a highly sensitive test is preferred, as it is better to identify as many frail individuals as possible, rather than to miss those who are actually frail. Considering our low sensitivity across all scales, the used cut-off points of specific scales can be changed. A strategy for maximizing the feasibility of frailty screening would be to conduct a stepwise process of increasingly more detailed assessment, that is, to combine the existing frailty scales for a comprehensive geriatric assessment. In addition, as good frailty scales should also be simple to apply, another consideration when choosing a scale is the time that is needed to complete it. FRAIL has the advantage of being easy and quick to administer, score and interpret. Conversely, while FI and TFI provide broader coverage of deficits and allow for better identification of high-risk individuals, they are more time-consuming. Thus, we suggest that while FI performs best for estimating risk, the FRAIL scale may be more practical to apply in the Chinese community-dwelling population. However, the increasing use of electronic health records (e.g., general practices) enables ready access to health measures across multiple domains. Then, both FI and TFI can be easily used as screening tools.

Strengths of this research include the longitudinal cohort design, a large, well-defined population-based sample, a wide range of baseline age, and a repeated comprehensive set of health-related assessments. These enable the operationalization and comparison of these four scales in the same Chinese population within the same timeframe.

Our study also has several potential limitations. A potential limitation is the reliance on self-reported questionnaires. We cannot rule out recall bias (e.g., regarding hospitalization over the past 4 years). Furthermore, the scales used here were adapted from the original definitions to utilize the data available from SAGE in shanghai, some important aging-specific variables, therefore, were not included, which may have influenced how each scale predicted outcomes. In particular, we used the lowest BMI for self-reported weight loss, which may have modified the scale characteristics, although this modification has been used previously in many studies. The modified measures, however, may be advantageous. For example, our measure of memory performance (assessed using a verbal recall test instead of a single self-reported question) can predict functional decline (38) and, unlike the self-reported memory used in the original scale, relies on objective assessment. A third limitation is that those who were excluded from the analyses of a scale due to missing data had a higher proportion of 4- and 7-year mortality (see Supplementary material S3). This study may slightly underestimate the ability of scales to predict all-cause mortality. Future studies could focus on verifying the usefulness of our operational approaches to frailty by replicating and extending our findings in other populations and settings.

## 5. Conclusion

In conclusion, we found that different approaches to frailty result in different estimates for the prevalence of at-risk individuals. Frailty defined using FI, FP, FRAIL, and TFI was independently associated with 4-year disability (except FP), hospitalization (except FP), and 4- and 7-year all-cause mortality in Chinese community-dwelling older adults. However, only FI, FRAIL and TFI were able to reliably predict these outcomes (except 4-year hospitalization), with fair-to-moderate predictive accuracy. Moreover, all four scales, while performing well at ruling out high-risk groups through high specificity estimates, were likely to miss large numbers of frail individuals as measured by adverse outcomes due to low sensitivity estimates. According to our study, FI performs best in estimating risk, while TFI and FRAIL are additionally useful, especially the latter, as a simple and faster screening tool, which may be more practical to apply in Chinese community-dwelling older adults either for the screening or diagnosis of frailty.

## Data availability statement

The raw data supporting the conclusions of this article will be made available by the authors, without undue reservation.

## Ethics statement

The studies involving human participants were reviewed and approved by Shanghai Center for Disease Control and Prevention Ethical Review Committee. The patients/participants provided their written informed consent to participate in this study.

## Author contributions

FQ did the statistical analyses, conducted the literature search, and wrote the first draft of the manuscript. All authors critically revised the manuscript for important intellectual content, approved the final version, and contributed to the study concept and design, acquisition, analysis, or interpretation of data.
