# Supplementary material for: Frailty and risk of adverse outcomes among community-dwelling older adults in China: a comparison of four different frailty scales
Source: Front Public Health. 2023 May 10;11:1154809. doi: 10.3389/fpubh.2023.1154809 (PMC10206323; doi:10.3389/fpubh.2023.1154809)
Supplement: Supplementary file 1 [file Table_1.DOCX]

| **Supplementary Material S1.** Baseline characteristics between responders and non-responders. | | | |
| --- | --- | --- | --- |
|  | Responders (n=3485) | Non-responders (n=1592) | p^†^ |
| Mean (SD) |  |  |  |
| Age (years) | 65.3 (8.6) | 65.8 (9.9) | 0.067 |
| BMI (kg/m^2^) | 19.6 (3.0) | 19.4 (2.9) | 0.008 |
| Number (%) |  |  |  |
| Sex (male) | 1589 (45.6) | 756 (47.5) | 0.210 |
| Marital status |  |  | 0.048 |
| Not partnered | 425 (12.2) | 226 (14.2) |  |
| Partnered | 3060 (87.8) | 1366 (85.8) |  |
| Educational level |  |  | 0.768 |
| No education | 632 (18.1) | 288 (18.1) |  |
| Less than primary | 468 (13.4) | 201 (12.6) |  |
| Primary | 670 (19.2) | 307 (19.3) |  |
| Secondary | 1002 (28.8) | 483 (30.3) |  |
| Higher | 713 (20.5) | 313 (19.7) |  |
| Smoking status |  |  | 0.423 |
| Never smoked | 2484 (71.3) | 1106 (69.5) |  |
| Former smoker | 145 (4.2) | 70 (4.4) |  |
| Current smoker | 856 (24.5) | 416 (26.1) |  |
| Frailty status (frail)^*^ |  |  |  |
| FI | 490 (14.1) | 244 (16.1) | 0.072 |
| FP | 411 (11.8) | 174 (11.7) | 0.945 |
| FRAIL | 124 (3.6) | 44 (2.9) | 0.219 |
| TFI | 209 (6.0) | 101 (6.7) | 0.384 |
| Abbreviations: SD=Standard Deviation; BMI=Body Mass Index; FI=Frailty Index; FP=Frailty Phenotype; TFI=Tilburg Frailty Indicator. | | | |
| †p value for comparison of difference between responders and non-responders: t-test or Wilcoxon rank-sum test (depending on distribution) for continuous variables, Chi-square test for categorical variables. | | | |
| *Due to missing data, small differences between n and numbers of participant reported for each scale can occur. | | | |
