# Supplementary material for: Frailty and risk of adverse outcomes among community-dwelling older adults in China: a comparison of four different frailty scales
Source: Front Public Health. 2023 May 10;11:1154809. doi: 10.3389/fpubh.2023.1154809 (PMC10206323; doi:10.3389/fpubh.2023.1154809)
Supplement: Supplementary file 2 [file Table_2.DOCX]

| **Supplementary Material S2.** An overview of all items constructed in each frailty scale. | |
| --- | --- |
| **Supplementary Material S2A.** List of the 35 variables included in the frailty index (FI) and cut points by domain. | |
| Variables | Deficits |
| **General health (1)** | |
| Self-rated health | Very good = 0; Good = 0.25; Moderate = 0.5; Bad = 0.75; Very bad = 1 |
| **Medically diagnosed conditions (9)** | |
| Arthritis; stroke; angina; diabetes; COPD; asthma; depression; hypertension; cataracts | No = 0; Yes = 1 |
| **Medical symptoms (6)**. In the last 30 days, how much. . .? | |
| ...of bodily aches or pains did you have? | None = 0; Mild = 0.25; Moderate = 0.5; Severe = 0.75; Extreme/cannot = 1 |
| ...of a problem did you have with sleeping? |  |
| ...difficulty did you have in seeing (person or object) across the road? |  |
| ...difficulty did you have in seeing an object at arm’s length? |  |
| ...difficulty did you have in hearing someone from the other side of the room? |  |
| ...difficulty did you have in hearing the content of talking? |  |
| **Functional activities assessments (11)**. In the last 30 days, how much difficulty did you have in... | |
| ...staying for a few days? | None = 0; Mild = 0.25; Moderate = 0.5; Severe = 0.75; Extreme/cannot = 1 |
| ...standing for long periods? |  |
| ...concentrating on doing something for 10 minutes? |  |
| ...walking 1 kilometer? |  |
| ...getting out of your home? |  |
| ...be emotionally affected by health condition? |  |
| ...joining in community activities? |  |
| ...dealing with daily work? |  |
| ...carrying things? |  |
| ...getting where you want to go? |  |
| **Cognitive function assessments (5)**. | |
| Immediate verbal recall. Number of words recalled correctly Trial 1 ,2 and 3. |  |
| Delayed Verbal Recall. Number of words recalled correctly. |  |
| Verbal Fluency. Total score (number of animals named correctly). |  |
| Forward Digit Span. Total score (the series number in the longest series repeated without error). |  |
| Backward Digit Span. Total score (the series number in the longest series repeated without error). |  |
| **Body mass index (BMI) (1)** |  |
| BMI [weight/(height2)]. Lowest quintile of body mass index (BMI)? | No = 0; Yes = 1 |
| **Physical performance tests (2)** |  |
| Grip strength. Grip (in Kg) (Left hand + Right hand)/2. Lowest quintile of grip strength stratified by sex and BMI, country-specific? | No = 0; Yes = 1 |
| Gait speed. Time (s) at normal/usual pace over 4 m. Lowest quintile of gait speed (walking 4 m) stratified by sex and height? | No = 0; Yes = 1 |

| **Supplementary Material S2B.** Frailty Phenotype (FP) Scale. | |
| --- | --- |
| Variables | Deficits |
| **Slowness:** The slowness was defined by the lowest quintile of gait speed (measured by recording the time taken in seconds to walk 4 meters at a normal/usual pace) stratified by sex and height as the following groups.  Man: height ≤173 cm, height >173 cm  Women: height ≤159 cm, height >159 cm | No = 0; Yes = 1 |
| **Weight loss:** The presence of the weight loss criterion was considered for the lowest quintile of body mass index (BMI). | No = 0; Yes = 1 |
| **Low grip strength:** The low grip strength was defined by the lowest quintile of grip strength (sum of highest values of two measurements on each hand) stratified by sex and BMI as the following groups.  Men: BMI ≤24, BMI 24-26, BMI 26-28, BMI >28  Women: BMI ≤23, BMI 23-26, BMI 26-29, BMI >29 | No = 0; Yes = 1 |
| **Exhaustion:** Individuals were asked “do you have enough energy for everyday life?” | Completely, Mostly, Moderately = 0; A little, Not at all = 1 |
| **Low physical activity:** The physical activity was assessed using the WHO Global Physical Activity Questionnaire (GPAQ). Individual who come had physical activity < 600 MET minutes a week were categorized for low physical activity. | No = 0; Yes = 1 |

| **Supplementary Material S2C.** FRAIL Scale. | |
| --- | --- |
| Variables | Deficits |
| **Fatigue:** Individuals were asked “do you have enough energy for everyday life?” | Completely, Mostly, Moderately = 0; A little, Not at all = 1 |
| **Resistance:** Individuals were asked “do you have any difficulty standing for long periods?” | None, Mild, Moderate = 0; Severe, Extreme/Cannot do = 1 |
| **Ambulation:** Individuals were asked “overall, in the last 30 days, how much difficulty did you have in walking a long distance such as 1 kilometer?” | None, Mild, Moderate = 0; Severe, Extreme/Cannot do = 1 |
| **Illness:** Individuals were classified as illness if they had 5 or more out of 9 self-reported diagnosis by asking “has a doctor ever told you that you had?”  (1) diabetes mellitus; (2) stroke; (3) cataracts; (4) angina pectoris; (5) arthritis; (6) asthma; (7) COPD; (8) depression; (9) hypertension | No = 0; Yes = 1 |
| **Weight loss:** The presence of the weight loss criterion was considered for the lowest quintile of body mass index (BMI). | No = 0; Yes = 1 |

| **Supplementary Material S2D.** Tilburg Frailty Indicator (TFI) Scale. | |
| --- | --- |
| Variables | Deficits |
| **Physical domains (8)** | |
| **Self-rated health:** Individuals were asked “in general, how would you rate your health today?” | Very good, Good, Moderate = 0; Bad, Very bad = 1 |
| **Weight loss:** The presence of the weight loss criterion was considered for the lowest quintile of body mass index (BMI). | No = 0; Yes = 1 |
| **Difficulty in walking:** The criterion was defined by the lowest quintile of gait speed stratified by sex and height as the following groups.  Man: height ≤ 173 cm, height > 173 cm  Women: height ≤ 159 cm, height > 159 cm | No = 0; Yes = 1 |
| **Difficulty in maintaining balance:** Individuals were asked “overall, in the last 30 days, how much difficulty did you have with getting up from lying down?” | None, Mild, Moderate = 0; Severe, Extreme/Cannot do = 1 |
| **Poor hearing:** Individuals were asked “do you have hearing problem?” | No = 0; Yes = 1 |
| **Poor vision:** Individuals were asked “in the last 30 days, how much difficulty did you have in seeing and recognizing person/object across the road or an object at arm's length?” | None, Mild, Moderate = 0; Severe, Extreme/Cannot do = 1 |
| **Lack of strength in hands:** The criterion was defined by the lowest quintile of grip strength (sum of highest values of two measurements on each hand) stratified by sex and BMI as the following groups.  Men: BMI ≤ 24, BMI 24-26, BMI 26-28, BMI > 28  Women: BMI ≤ 23, BMI 23-26, BMI 26-29, BMI > 29 | No = 0; Yes = 1 |
| **Physical tiredness:** Individuals were asked “do you have enough energy for everyday life? | Completely, Mostly, Moderately = 0; A little, Not at all = 1 |
| **Psychological domains (4)** | |
| **Poor memory:** Verbal recall test (the investigator read 10 words to individuals and asked them to repeat these words as much as possible) was used to assess the memory. The presence of poor memory was defined by the lowest quintile of recall 10 nouns. | No = 0; Yes = 1 |
| **Feel down:** Individuals were asked “during the last 12 months, have you had a period lasting several days when you felt sad, empty or depressed? | No = 0; Yes = 1 |
| **Nervous or anxious:** Individuals were asked “during this period, did you feel anxious and worried most days?” | No = 0; Yes = 1 |
| **Cope with problems:** Individuals were asked “how often have you found that you could not cope with all the things that you had to do?” | Never, Almost never = 0; Sometimes, Fairly often, Very often = 1 |
| **Social domains (3)** | |
| **Live alone:** Individuals were asked “number of people living in household” | >1 = 0; 1 = 1 |
| **Family dysfunction:** Individuals were asked “how satisfied you are with your personal relationships?” | Very satisfied, Satisfied, Neither satisfied nor dissatisfied = 0; Dissatisfied, Very Dissatisfied = 1 |
| **Low social support:** Individuals were asked “do you receive the support from others when you need it? | Yes = 0; No = 1 |
