# Supplementary material for: Frailty and risk of adverse outcomes among community-dwelling older adults in China: a comparison of four different frailty scales
Source: Front Public Health. 2023 May 10;11:1154809. doi: 10.3389/fpubh.2023.1154809 (PMC10206323; doi:10.3389/fpubh.2023.1154809)
Supplement: Supplementary file 3 [file Table_3.DOCX]

| **Supplementary Material S3.** Adverse outcomes between included and excluded cases for each scale. | | | | | | | | | | | | | |
| --- | --- | --- | --- | --- | --- | --- | --- | --- | --- | --- | --- | --- | --- |
| Frailty scale | Included  Cases  (n (%)) | 4-year disability (%) | | | 4-year hospitalization (%) | | | 4-year mortality (%) | | | 7-year mortality (%) | | |
|  |  | Included  Cases | Excluded Cases | p^†^ | Included  Cases | Excluded Cases | p^†^ | Included  Cases | Excluded Cases | p^†^ | Included  Cases | Excluded Cases | p^†^ |
| FI | 5266 (97.5) | 3.6 | 0 | 0.279 | 20.6 | 37.5 | 0.122 | 5.3 | 33.1 | <0.001 | 8.6 | 45.6 | <0.001 |
| FP | 5158 (95.5) | 3.6 | 0 | 0.349 | 20.6 | 25.0 | 0.716 | 4.0 | 48.8 | <0.001 | 6.7 | 70.9 | <0.001 |
| FRAIL | 5299 (98.1) | 3.6 | 0 | 0.787 | 20.7 | 0 | 0.496 | 5.4 | 36.9 | <0.001 | 8.8 | 50.5 | <0.001 |
| TFI | 5272 (97.6) | 3.6 | 0 | 0.508 | 20.7 | 16.7 | 0.804 | 5.2 | 38.5 | <0.001 | 8.5 | 53.9 | <0.001 |
| Abbreviations: SD=Standard Deviation; BMI=Body Mass Index; FI=Frailty Index; FP=Frailty Phenotype; TFI=Tilburg Frailty Indicator. | | | | | | | | | | | | | |
| †p value for comparison of difference between outcome rates of included and excluded cases for each scale using Chi-square or Fisher exact test as appropriate. | | | | | | | | | | | | | |
